# *Allium cepa* as a Model System for Assessing the Phytotoxicity of Food Dye Mixtures: An Integrated Analysis of Growth Rates and Physiological Parameters

**DOI:** 10.3390/plants15131968

**Published:** 2026-06-26

**Authors:** Oana-Alexandra Găinaru, Daniela-Georgiana Ciobanu, Nicoleta Ianovici

**Affiliations:** 1 Biology Department, Faculty of Chemistry, Biology and Geography, West University of Timișoara, 300223 Timișoara, Romanianicoleta.ianovici@e-uvt.ro (N.I.); 2 Environmental Biology and Biomonitoring Research Center, The Institute for Advanced Environmental Research Timișoara, 300223 Timișoara, Romania

**Keywords:** *Allium cepa*, *Allium* test, phytotoxicity, food dyes, tartrazine E102, quinoline yellow E104, sunset yellow E110, carmoisine E122, brilliant blue E133

## Abstract

Synthetic food dyes represent an important category of chemical contaminants with potential phytotoxic, genotoxic, and ecotoxic effects on living organisms and natural ecosystems. The present study aimed to evaluate the physiological effects induced by three commercial food dyes on the model organism *Allium cepa*, using the *Allium* test under hydroponic conditions. A total of 105 bulbs were exposed for 48 h to two different concentrations of each dye, for which gravimetric and physiological parameters, such as biomass, water content, mineral and organic composition, growth inhibition index, tolerance index, and relative growth rate, were subsequently analyzed. Statistical analyses revealed significant differences between batches for all evaluated parameters, indicating effects mainly dependent on the type of dye applied. The results suggest that exposure to synthetic food dyes causes disturbances in water balance, biomass accumulation and mineral homeostasis, confirming the phytotoxic potential of these compounds and the utility of the *Allium* test in the biomonitoring of chemical contaminants.

## 1. Introduction

Anthropogenic activities have led to pollution of the natural environment in numerous forms, consciously or unintentionally, damaging the biosphere and thus the quality of life of all organisms [1]. The discovery and development of numerous compounds used to improve daily life have led to the introduction of new chemical substances, foreign to the biosphere, which are not necessarily found naturally in the environment or whose natural concentrations are significantly different from those caused by anthropogenic activity [2,3]. Most often, exposure of vulnerable organisms to these substances has shown genotoxic, cytotoxic, and mutagenic effects, inducing lesions in the genetic material that can be transmitted to descendants, thus affecting even future generations [4].

Due to their stationary nature and long-term exposure to contaminated environments, plants are among the most vulnerable organisms to xenobiotic pollution [5]. They can take up various compounds from the soil, water or atmosphere which, after entering the body, cause biochemical and physiological changes in response to their toxic action [6]. Contaminated fruits and vegetables are subsequently consumed by animals and humans, causing negative effects along the entire food chain [7].

Considering these aspects, cyto-genotoxicity and mutagenicity tests have been used in recent decades for the purpose of evaluating the potential adverse effects of pollutants, the risks of exposure to living organisms and monitoring the natural environment [8,9]. Since the direct assessment of the exposure of human organisms to various xenobiotics is not always feasible for ethical, logistical and practical reasons, a series of experimental models represented by upper plants have been proposed for this purpose [4]. Due to their ease of application and use, low cost, high sensitivity, ecological relevance and good correlation with animal systems [5,8], plant models such as *Allium cepa*, *Vicia faba*, *Zea mays*, *Tradescantia* sp., *Nicotiana tabacum*, *Crepis capillaris*, *Lactuca sativa* or *Hordeum vulgare* [10,11] are frequently used for genotoxicity assessment and monitoring, both in laboratory conditions and in the natural environment, of different chemical contaminants [4]. However, the responses evaluated for toxicity tests depend on the nature of the bioindicator used and the compound investigated, as well as on the physiological and/or genetic parameters evaluated [12].

Among the proposed plant models, *Allium cepa* has become one of the most frequently used model organism in toxicity and cytogenetic studies, due to its karyotype with a reduced number of chromosomes (2n = 16) characterized by their larger size, the rapid and continuous division of root meristems, the well-individualized mitotic phases and the presence of the oxidative enzyme system, essential in the evaluation of promutagenic effects [8,13,14].

The *Allium* Test (AT) was first introduced in 1938 by Levan in his studies on the effects of colchicine on mitosis [15] and later developed through contributions by Grant, Fiskesjö, Rank and Nielsen, who made the method more useful for environmental monitoring and for testing complex mixtures of substances [10,14]. It assesses toxic, cytotoxic, genotoxic and mutagenic effects by examining chromosomal alterations and DNA damage in root meristematic cells [16,17]. The usefulness of the test derives from its ability to simultaneously highlight effects at the cellular, tissue and systemic levels, allowing the identification of DNA lesions, chromosomal aberrations and disruptions of the mitotic process [18], thus providing relevant information on the mutagenic and carcinogenic potential of various targeted substances, such as drugs, medicinal plant extracts, pesticides, insecticides, herbicides, fungicides, sediments, surface and groundwater, industrial effluents, food additives, feed additives, heavy metals, nanomaterials, etc. [14,19,20]. Due to its simplicity and short application time, good correlation with other test systems and high efficiency, the *Allium* test is one of the most efficient and reliable biomonitoring tests for various xenobiotics, being recognized as such by the World Health Organization (WHO) and the US Environmental Protection Agency (EPA), being included in the International Plant Bioassay Program, the International Program for Chemical Safety and the United Nations Environment Program [9,13,21].

Methodologically, the test involves direct exposure of bulbs or germinated seeds of *A. cepa* to water, agar, soil or solution containing the tested substance, their roots being subsequently analyzed both microscopically and macroscopically [17]. The most important cytogenetic parameters calculated based on microscopic observations are represented by the mitotic index (MI), which reflects the cytotoxicity of the analyzed substance, the proportion of chromosomal abnormalities (CA), which shows the structural or numerical damage of the chromosomes, the presence of nuclear aberrations (NA), the proportion of micronuclei (MN), which indicates mutagenic effects and chromosome segregation errors, the mitotic phases index (IP), as well as the cytotoxicity limit value (LCV) [10,14,17].

Regarding the macroscopic aspects, the most frequently used parameters are represented by the root growth length (RGL—average root length for each sample), based on which the percentage of inhibition of root growth in the tested extracts compared to the control can be calculated. In addition, the EC_50_ (the effective concentration at which root growth is 50% of the control) can be calculated. At the same time, changes in root appearance can be evaluated to estimate the toxicity index (color, consistency, turgor, root tip shape, presence of malformations or necrosis) [14,22]. Moreover, in extended versions of the test, gravimetric parameters (biomass, water content, mineral composition, etc.) [22,23,24,25], physiological parameters (germination rate, survival rate, root length, tolerance index, etc.) [19,26,27] and biochemical parameters (activity of antioxidant enzymes such as superoxide dismutase, catalase, ascorbate peroxidase, glutathione, etc., as well as lipid peroxidation, H_2_O_2_ production, total soluble proteins, etc.) [27,28] can also be included, which allows the correlation of cytogenetic effects with the growth and metabolism responses of the plant.

Food dyes are a category of chromophoric chemical substances used in the food industry as additives capable of imparting, intensifying or restoring the color of food and beverages, independently or following chemical reactions with other compounds [29,30]. They include both natural constituents of food and substances of plant, animal or mineral origin, as well as synthetic or semi-synthetic compounds obtained by chemical complexation of natural substances or by chemical synthesis of artificial compounds, structurally identical to natural pigments [31]. According to the European framework, substances obtained by physico-chemical extraction from edible raw materials are also considered as coloring agents when the extraction is selective for pigments over nutritional or aromatic components [32].

The use of dyes as food additives is regulated and monitored by a set of laws and control institutions, with a role in ensuring consumer safety and the responsible use of these substances [33,34,35,36]. Only additives approved for consumption can be used in food products, and their application must comply with requirements such as technological necessity, absence of health risks according to scientific evidence and avoidance of misleading consumers [37,38,39]. Currently, in the European Union, the use of 41 food dyes [35,40] is permitted, whose presence must be indicated on the food label by mentioning the specific European indicator and/or the name of the colorant [41,42,43].

The visual impact of food is the first way in which people experience it, long before they perceive the aroma or taste of the products they choose to consume [35]. Thus, its color is able to influence both product recognition and acceptability, colorants being used to make food more attractive and stimulate appetite, especially in products such as desserts, sweets, juices and soft drinks [41,44]. The food industry also aims to harmonize color with consumer expectations, as deviations from their preconceived idea of how the products should look can divert their purchasing power [35,40]. This reasoning is exploited especially in the case of products intended for children and young people, where bright shades and multiple colors are used to increase their attractiveness, children being particularly sensitive to visual stimuli and preferring intensely colored drinks, cereals, sweets or ice creams [44,45].

Since their appearance, the food industry has relied on the use of synthetic dyes to meet the visual needs of consumers. Among them, azo dyes such as Tartrazine (E102), Sunset Yellow (E110), Carmoisine (E122), Ponceau Red 4R (E124), Allura Red/Red 40 (E129), Brilliant Blue (E133), Brown HT (E155), etc., represent the largest class of synthetic dyes, estimated at over 60% of world production [46]. Due to their high stability to light, heat, pH variations or processing technologies (pasteurization, baking, food storage), as well as their increased solubility [47], they are able to reproduce a wide range of vibrant colors and keep them constant over time [48], to the detriment of natural pigments that are labile over time, to variations in light, pH or temperature and show variability and intensity of colors dependent on the harvests obtained [30].

Synthetic dyes are economical and versatile; their synthesis processes, starting from petrochemical precursors, present high yields and much lower costs per unit of color compared to their natural equivalents [49]. Moreover, due to the wide spectrum of utility areas—being involved not only in the food industry, but also in the manufacturing processes of beverages, medicines, supplements, cosmetics, textiles, paper, etc. [50]—and due to the ease of their formulation and quality control, based on their well-defined chemical structures, the category of synthetic pigments still represents the most rational option, despite the toxicological risks that are gradually pushing the industry towards natural alternatives [30,51].

Suspicions about the potential adverse effects of the consumption of these food additives have existed since the beginning of the 20th century [52], and the potential impact on human health has been intensively researched to date. The most frequently reported adverse effects are hypersensitivity reactions and food allergies [53]. Certain azo dyes, such as Tartrazine (E102), Sunset Yellow (E110), Carmoisine (E122), Amaranth (E123), Ponceau Red 4R (E124) or Allura Red (E129), can trigger allergic reactions such as urticaria, pruritus, skin rashes, angioedema or exacerbation of asthmatic symptoms, especially in people with atopic predisposition or those with aspirin intolerance [54]. Although the European Food Safety Authority (EFSA) estimates that such reactions occur in a relatively small proportion of the population, they have been clinically documented and represent an important concern in the assessment of the safety of food additives [40,55].

Studies on the neurobehavioral effects of food dyes have shown a negative correlation between the consumption of mixtures of synthetic dyes (E102, E104, E110, E122, E124 and E129) in combination with sodium benzoate (E211) and increased levels of hyperactivity or concentration difficulties in children [41,45,56,57]. For this reason, starting in 2008, the presence of additional information for warning purposes is mandatory on the label of food products containing any of the six dyes listed above, in the form of statements such as *“may adversely affect the activity and attention of children”* [32,55,58].

Numerous experimental studies have demonstrated the genotoxic and mutagenic potential of azo dyes, compounds such as E102, E110, E123 or E124 inducing oxidative stress, DNA damage, changes in gene expression involved in apoptosis and genetic material repair processes, micronuclei formation or various chromosomal aberrations in in vitro experiments and animal models [33,59]. These effects have been associated with the intestinal metabolism of dyes, where the microbiota has the ability to reduce them to the aromatic amine stage, which has a known carcinogenic and mutagenic potential [33,59]. Moreover, some commercial preparations may contain traces of potentially carcinogenic impurities, such as benzidine or β-naphthylamine, even though these are strictly limited by regulations [40,60]. Despite the results present in the literature, the most recent assessments by regulatory agencies concluded that the carcinogenic risk is not demonstrated at current levels of use [40,61]. Chronic exposure to various types of synthetic dyes has been associated with biochemical and histological changes in the liver and kidneys, increases in liver enzymes, oxidative stress, changes in lipid profiles and hematological parameters, gastrointestinal disorders such as nausea or diarrhea, as well as changes in the activity of some digestive enzymes [45,62,63,64]. Effects on the reproductive and immune systems, including reduced sperm count and testicular damage, have also been reported in experimental exposures at high doses [65].

An important aspect in assessing the risks associated with food dyes is the simultaneous exposure to multiple additives. In real-life conditions, humans are frequently exposed to complex mixtures of colors and preservatives, experimental studies suggesting that these combinations may generate cumulative or synergistic effects [41]. Most toxicological assessments and acceptable daily intake (ADI) limits are established for each compound individually, which may underestimate the risks for certain sensitive groups, especially children [57]. Although natural colors are generally considered safer than synthetic ones, they are not completely risk-free. Some natural substances can cause allergic reactions or contain contaminants from the extraction process or the environment of origin [29,41]. Also, certain degradation products resulting from food processing can have undesirable biological effects [40].

The environmental impact of synthetic food dyes is well documented, despite the fact that public discourse focuses more on the risks directly affecting consumers. Every year, tremendous quantities of dyes and pigments are produced globally (for food, textiles, cosmetics industries, etc.), a significant portion of which is lost during technological processes or discharged as effluents during industrial use [66,67]. Synthetic dyes, especially azo dyes, are composed of complex aromatic molecules that present azo functional groups and sulfonic substituents, which give them high stability to light, temperature and oxidation, being difficult to biodegrade and refractory to common biological treatment processes [66,68].

The presence of industrial dyes alters the physicochemical properties of clean water, changing its pH and giving it an intense color, reducing transparency and light penetration, which affects the photosynthesis processes of aquatic plants and algae, and leads to a decrease in the concentration of dissolved oxygen. Thus, the self-healing capacity of aquatic ecosystems is compromised, exacerbating eutrophication processes, vertebrate death and imbalances in aquatic communities [66]. In addition to optical and physicochemical effects, dyes and their degradation products are often toxic, genotoxic and mutagenic for aquatic and terrestrial organisms [68]. Azo dyes are reduced by anaerobic microbiota in sediments and in the digestive systems of animals to the stage of aromatic amines, compounds well known as mutagens and carcinogens, which can form DNA adducts [45].

The complexity of the problem is accentuated by the fact that the degradation of dyes in the environment does not lead to their abolition, but can generate intermediates with much higher toxic potential than the initial compound [67]. Photodegradation and chemical transformations of dyes in water lead to the appearance of products with variable biodegradability and toxicity, while some advanced oxidation processes (AOP) or chemical treatments (hypochlorite, ozone, UV/NaOCl) can rapidly decolorize effluents, but can increase their toxicity by forming partially oxidized species [66,68,69].

The environmental impact of synthetic dyes is not limited to the food sector, as the same dyes are also widely used in the textile, paper, cosmetics and pharmaceutical industries, which means that effluents from multiple sources accumulate in the same watershed [66]. In developing countries, where treatment infrastructure is insufficient and enforcement of regulations is difficult, the direct discharge of contaminated water into rivers and lakes causes severe degradation of water quality, with direct and indirect effects on human health (through drinking water, irrigation, fish consumption, etc.) and on the integrity of ecosystems [41].

Taking into account all the aspects presented above, the present work aims to assess some of the physiological parameters characteristic of the *Allium* test, in order to evaluate the phytotoxic effects of solutions containing varying proportions of food dyes, on plant organisms grown under hydroponic conditions, thus emulating plausible situations of contamination of groundwater with mixtures of such chemicals.

## 2. Results

### 2.1. Gravimetric Data Analysis

**Figure 1:**
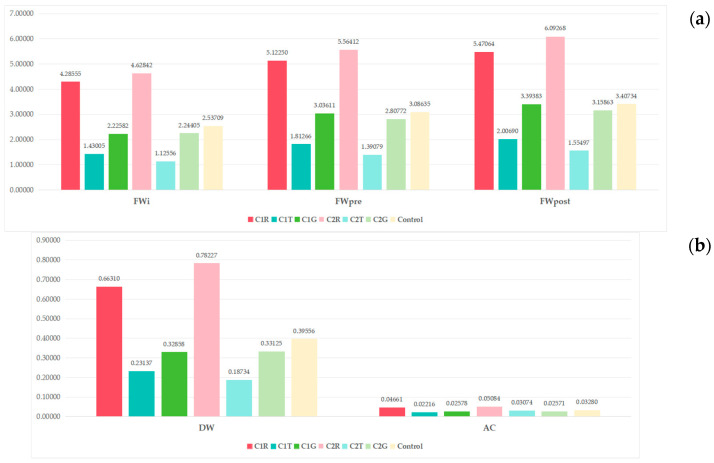
The mean values of the three fresh biomasses determined (FWi, FWpre and FWpost) (**a**), the dry biomass (DW) and ash content (AC) (**b**) for the seven analyzed lots.

The results of the primary statistical analysis performed for the gravimetric parameters obtained from the five weighings performed during the experiment are found in Table 1 and a graphical representation of their mean values is shown in Figure 1.

The Shapiro–Wilk normality test revealed a predominantly normal distribution, except for the data related to the initial biomass corresponding to the lot treated with the C1 turquoise dye solution and the dry biomass corresponding to the lot treated with the C1 red dye solution. The Levene test revealed a non-homogeneous distribution of the data related to all 5 gravimetric parameters. These results indicate the presence of large variations between individuals or extreme values in the case of the respective lots, which may suggest a heterogeneity of the biological response to some of the treatments applied.

The analysis of variance revealed significant differences in all analyzed parameters, with a large effect size in all 5 cases (ε^2^ > 0.14), the results being presented in Table 2. Post hoc analysis revealed significant differences between most batches in the case of initial biomass, except for the comparison between lots treated with solution C1 and homologues treated with solution C2 for each dye separately, as well as between both batches related to the green dye and the control lot.

The observed differences were also perpetuated in the case of wet biomasses pre- and post-treatment. In the case of dry biomass, significant differences were observed between the analyzed batches, except for the comparisons between the two solutions of each dye separately, between the lot treated with solution C1 of the turquoise dye and the lot treated with solution C2 of the green dye, and between both batches related to the green dye and the control. Regarding the ashes, insignificant exceptions were observed between the red dye lots, between the lot treated with the C1 solution of the turquoise dye and the green dye lots, between the lot treated with the C2 solution of the turquoise dye, the lot treated with the C2 solution of the green dye and the control, as well as between the lot treated with the C1 solution of the green dye and the lots treated with the C2 solutions of the turquoise and green dyes. The results of the Dunn test suggest a physiological reaction dependent on the type of dyes tested, but independent of their concentration in terms of the growth and accumulation processes of biomass, the turquoise dye presenting significant differences between the two concentrations tested in terms of the accumulation of inorganic matter in the bulbs, while the green dye demonstrated a significant influence in the case of both concentrations tested compared to the control lot, indicating a significant influence even at low concentrations.

**Figure 2:**
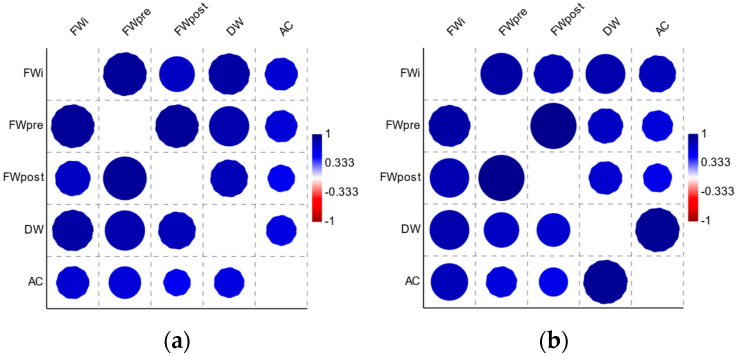
Correlations of gravimetric parameters related to batches treated with concentration 1 (**a**) and, respectively, concentration 2 (**b**) of the red dye solutions. The correlation coefficients are represented by the colored dots inside the matrix, their size and color illustrating the nature of the correlation (the bigger the dot, the closer the resulting value is to 1. Blue represents a positive correlation, varying from almost white when the value is closer to 0, up to deep blue when the value is closer to 1. Similarly, red represents a negative correlation, varying from almost white when the value is closer to 0, up to dark red when the value is closer to 1. The levels of strength of association considered are very strong correlation (±0.80 to ±1.00), strong correlation (±0.60 to ±0.79), moderate correlation (±0.40 to ±0.59), weak correlation (±0.20 to ±0.39) and very weak or negligible correlation (0.00 to ±0.19). For *p*-values > 0.05, correlation dots are crossed out.

The analysis of the interdependence of the gravimetric parameters was performed for each batch separately by calculating the Spearman correlation coefficient. In the case of the batch treated with the red dye solution C1, very strong positive correlations were observed between FWi and the FWpre (rs = 0.91429), FWi and DW (rs = 0.86786), FWpre, FWpost (rs = 0.91071) and DW (rs = 0.83929). Strong positive correlations were observed between FWi, FWpost (rs = 0.74286) and AC (rs = 0.68929), between FWpre and AC (rs = 0.675), as well as between DW and AC (rs = 0.63571), with a moderate positive correlation between FWpost and AC (rs = 0.56786) (Figure 2).

In the case of the batch treated with the red dye solution C2, very strong significant correlations were observed between FWi, FWpre (rs = 0.87857), FWpost (rs = 0.82418) and DW (rs = 0.83297), between FWpre and FWpost (rs = 0.96484) and between DW and AC (rs = 0.92967). Strong positive correlations were observed between FWi and AC (rs = 0.78022), FWpre, DW (rs = 0.74066) and AC (rs = 0.65275) and between FWpost, DW (rs = 0.7011) and AC (rs = 0.6044) (Figure 2).

**Figure 3:**
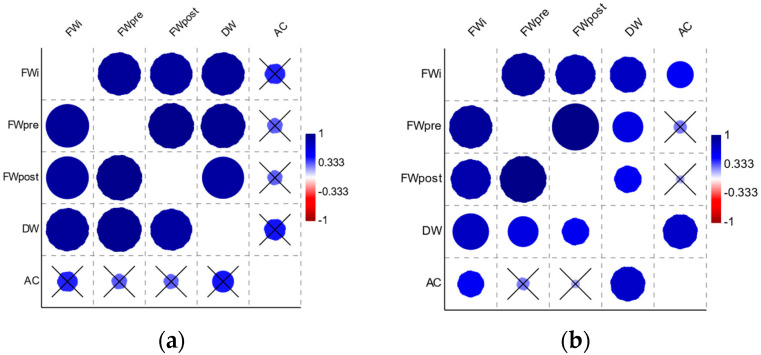
Correlations of gravimetric parameters related to batches treated with concentration 1 (**a**) and concentration 2 (**b**) of the turquoise dye solutions, respectively.

Very strong positive correlations were observed between FWi, FWpre (rs = 0.91071), FWpost (rs = 0.89643) and DW (rs = 0.91071), between FWpre, PWpost (rs = 0.96429) and DW (rs = 0.94286) and between FWpost and DW (rs = 0.88214), the rest of the parameters showing a statistically insignificant correlation in the case of the batch treated with the turquoise dye solution C1 (Figure 3).

In the case of the batch treated with the turquoise dye solution C2, very strong positive correlations were observed between FWi, FWpre (rs = 0.90357) and FWpost (rs = 0.83929) and between FWpre and FWpost (rs = 0.97857) (Figure 3). Strong positive correlations were observed between FWi and DW (rs = 0.75714), between FWpre and DW (rs = 0.63571) and between DW and AC (rs = 0.72857). Moderate positive correlations were observed between FWi and AC (rs = 0.55357) and between FWpost and DW (rs = 0.56786), the rest of the relationships being statistically insignificant.

**Figure 4:**
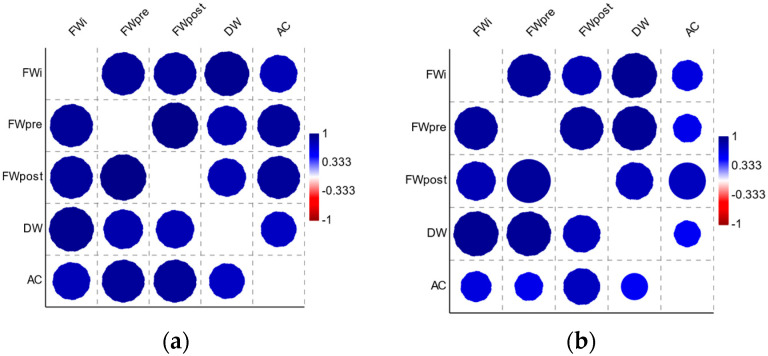
Correlations of gravimetric parameters related to batches treated with concentration 1 (**a**) and concentration 2 (**b**) of the green dye solutions, respectively.

Very strong positive correlations were observed in all relationships between the gravimetric parameters related to the batch treated with the C1 solution of the green dye, except for FWi and AC (rs = 0.79643) and DW and AC (rs = 0.75357), respectively, among which there were strong positive correlations (Figure 4).

In the case of the batch treated with the C2 green dye solution, very strong positive correlations were observed between FWi, FWpre (rs = 0.8989), FWpost (rs = 0.81538) and DW (rs = 0.93407), as well as between FWpre, FWpost (rs = 0.9033) and DW (rs = 0.92527) (Figure 4). Strong positive correlations were present between FWi and AC (rs = 0.63956) and between FWpost, DW (rs = 0.78022) and AC (rs = 0.76264). There is a moderate positive correlation between AC, FWpre (rs = 0.59121) and DW (rs = 0.55165).

**Figure 5:**
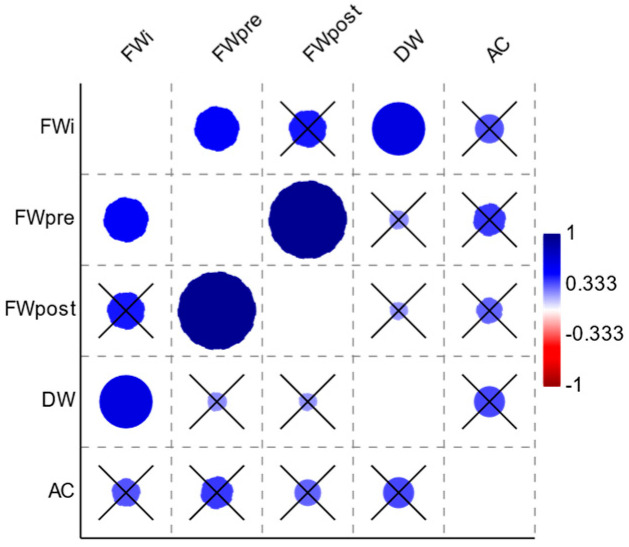
Correlations of gravimetric parameters related to the control lot.

In the case of the control group, the only statistically significant correlations observed were between FWi, FWpre (rs = 0.53571) and DW (rs = 0.63214) and between FWpre and FWpost (rs = 0.94286) (Figure 5).

### 2.2. Physiological Parameters Results

**Figure 6:**
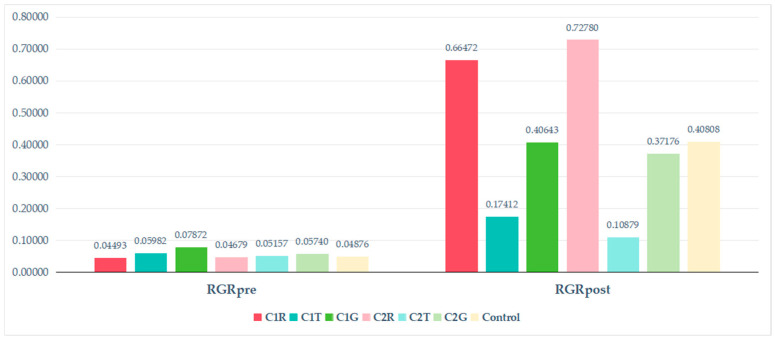
The mean values of pre- and post-treatment relative growth rates (RGRpre and RGRpost) for the seven analyzed lots.

**Figure 7:**
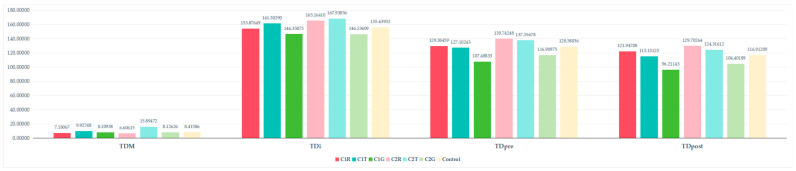
The mean values of tissue mineral deposition (TDM), initial, pre- and post-treatment tissue density (TDi, TDpre and TDpost) for the seven analyzed lots.

**Figure 8:**
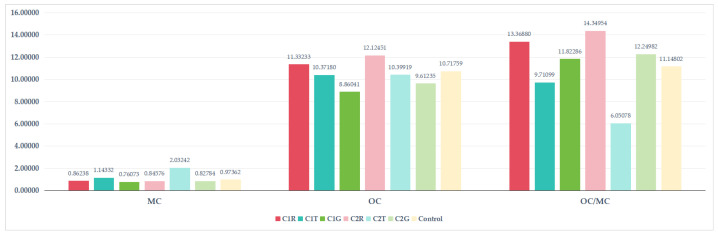
The mean values of mineral and organic content (MC and OC) and the ratio between the two parameters for the seven analyzed lots.

**Figure 9:**
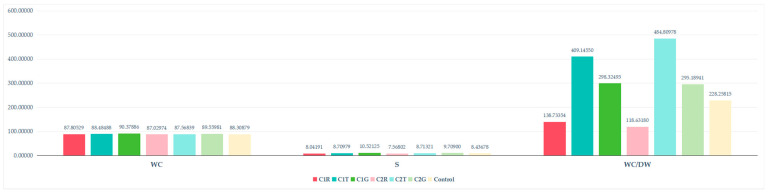
The mean values of water content (WC), succulence (S) and the ratio between the water content and dry biomass (WC/DW) for the seven analyzed lots.

**Figure 10:**
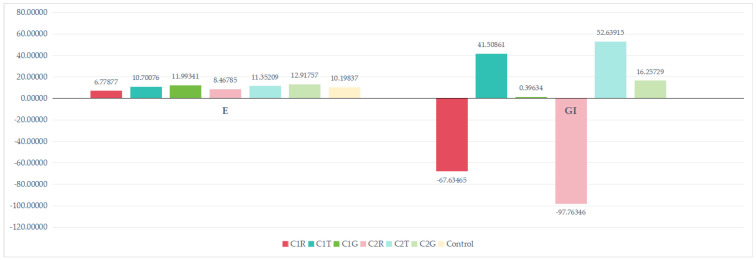
The mean values of biomass accumulation efficiency index (E) and growth inhibition index (GI) for the seven analyzed lots.

**Figure 11:**
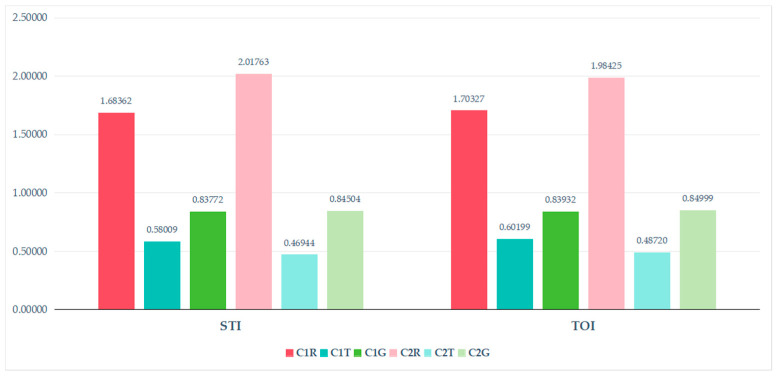
The mean values of the stress tolerance factor and tolerance index for the seven analyzed lots.

Physiological parameters described in the experimental methodology, calculated based on the five gravimetric parameters presented previously, were analyzed for the purpose of their statistical characterization, with the graphical representation of their mean values being shown through Figure 6, Figure 7, Figure 8, Figure 9, Figure 10 and Figure 11.

The Shapiro–Wilk test revealed a predominantly normal distribution, with significant exceptions in the case of certain groups in each of the 13 parameters concerned. The Levene homogeneity test highlighted the predominantly inhomogeneous distribution of the data, maintaining the hypothesis of the presence of a significant variation between the analyzed data or of a heterogeneous response of the organisms to the applied treatments.

The analysis of variance revealed significant differences between groups for all determined physiological parameters, the effect size being predominantly large (ε^2^ > 0.14 for 16 out of 21 parameters) or moderate (ε^2^ < 0.14 for 4 out of 21 parameters) in all cases except for biomass accumulation efficiency, for which the effect size is small (ε^2^ < 0.06) (Table 3).

Post hoc analysis revealed significant differences between the lot treated with the C1 solution of the green dye and all other compared lots, except for the lot treated with the solution C1 of the turquoise dye, in the case of RGR pre-treatment. In the case of RGR post-treatment, significant differences were observed in the vast majority of the compared cases, with the exception of the lots treated with the C1 solutions of each dye and the groups treated with the homologous C2 solutions, as well as between both batches treated with the green dye and the control lot. The comparison of the average results obtained for each batch highlights a relative growth trend between the pre- and post-treatment moments (Figure 6), which, together with the Dunn test results, indicates an independent influence of concentration manifested by the red and turquoise dyes on the growth processes, the green dye solutions insignificantly affecting the development of plants following the application of the treatments.

Mineral deposition at the tissue level showed significant differences in the vast majority of comparisons made in the case of post hoc analysis, highlighting a heterogeneous biological response, depending on the treatments applied. The observed exceptions were between the lot treated with the C1 red dye solution, the homologous lot treated with the solution C2, the batches related to the green dye and the control, between the lot treated with the C1 turquoise dye solution, the lot treated with the C1 green dye solution and the control, as well as between the batches related to the green dye, the comparison between the two and compared to the control lot. In the case of the batches treated with the C2 solutions of the red and turquoise dyes, significant differences were observed compared to the control batch, which may argue that the exposure of the bulbs to these substances causes significant changes in the tissue mineral composition, reflecting possible disturbances in ionic homeostasis and associated metabolic processes.

The tissue density of the analyzed bulbs, determined comparatively according to all three types of wet biomass, shows a uniform tendency to decrease in tissue mass in the case of all the analyzed batches (Figure 7), which can be correlated with the results of normal physiological processes of water accumulation between the three points in time analyzed in the experiment. TD showed significant differences between the batches treated with the green dye solutions and all other batches, except for the control batch, in the case of tissue density determined based on initial biomass. The observed differences were maintained within the three instances of the parameter, suggesting an adaptive physiological response to the stress caused by the applied treatments, in the case of bulbs treated with green dye.

Dunn’s test revealed significant differences between the batches treated with the turquoise dye solutions and all other batches except for the comparison between the two and the control lot in relation to the lot treated with the C1 turquoise solution. Significant differences also exist between the batches related to the green dye and the control batch in the case of mineral content. In the case of organic content, significant differences were observed in the batches related to the green dye and all other lots except for the control lot in relation to the lot treated with the C2 green dye solution, as well as between the lot treated with the C2 red dye solution and the one treated with the C2 turquoise dye solution. The ratio of the two parameters showed significant differences between the batches treated with solutions C2 of the red and turquoise dyes and all other batches except for the comparison between the two lots related to the red dye, as well as between the lot treated with C1 solution of the red dye and the lot treated with C1 solution of the turquoise dye. Taking into account the analysis of the gravimetric parameters, the results illustrate the metabolic imbalances induced by the treatments and dependent on their composition, especially in the case of red and turquoise dyes, while also arguing the influence of the green dye on the processes of accumulation of organic substances at high concentrations, while the turquoise dye demonstrates a disruptive action on the processes of accumulation of mineral substances at low concentrations, the compensatory reaction being associated with osmotic stress or metabolic imbalances.

Water content and demonstrated the same statistical differences, observed between the batch treated with the C1 concentration of the green dye and all other batches analyzed, while, in the case of the ratio between water content and dry biomass, significant differences were observed in all batches analyzed except for the comparison between batches treated with solutions C1 and C2 of the same dye and between the batch treated with solution C2 of the green dye and the control. Results confirm the presence of systemic effects on water balance manifested by the treatments applied and dependent on the administered concentration, the presence of significant differences in terms of metabolic response, especially in the case of high concentrations, which can be interpreted as a compensatory response to stress, associated either with the accumulation of water in tissues or with the reduction in the proportion of dry matter, a phenomenon correlated with the decrease in tissue density previously observed.

Water content and succulence demonstrated the same statistical differences present between the lot treated with the C1 concentration of the green dye and all other batches analyzed, and between the lot treated with the C2 solution of red dye and the lot treated with the C2 solution of green dye. In the case of water content, significant differences were also noted in the case of the lot treated with the C2 solution of the green dye and the batches treated with the C1 solution of the red dye and, respectively, the C2 solution of the turquoise dye, while, in the case of the ratio between water content and dry biomass, significant differences were observed in all batches analyzed except for the comparison between the batches treated with the C1 and C2 solutions of the same dye, between the lot treated with the C1 solution of the turquoise dye and the lot treated with the C2 solution of the green dye, as well as between the lot treated with the C2 solution of the green dye and the control. Results confirm the presence of systemic effects on water balance manifested by the treatments applied and dependent on the concentration administered, the presence of significant differences in metabolic response, especially in the case of high concentrations, potentially being interpreted as a compensatory response to stress that can be associated either with the accumulation of water in tissues or with the reduction in the proportion of dry matter, a phenomenon correlated with the decrease in tissue density previously observed.

Dunn’s test applied to the efficiency of biomass accumulation demonstrated significant differences between the lot treated with the C1 solution of the red dye and all the batches analyzed, except for the lot treated with the homologous C2 solution and the control lot, as well as between the lot treated with the C2 solution of the red dye, the lot treated with the C1 solution of the green dye and the batches treated with the C2 solutions of the other dyes. The growth inhibition index, as well as the stress tolerance factor and the tolerance index revealed the same significant differences between all the batches analyzed, except for the situation in which the C1 and C2 solutions of the same dye were compared and in the case of the comparison between the lot treated with the C1 solution of the turquoise dye and the lot treated with the C2 solution of the green dye. The results illustrate an adaptive physiological response to biological stress induced by the treatments, dependent on the nature of the dye, but independent of the applied concentration, with phytotoxic and growth inhibitory effects being evident for all of the applied dyes.

## 3. Discussion

The statistical analysis demonstrated significant differences amongst all the gravimetric and physiological parameters employed in the current study, suggesting overall, a high biological variability and probability of a heterogeneous response of the *Allium cepa* bulbs to the applied treatments. The analysis of variance indicated significant differences between batches for all analyzed parameters, with predominantly large effect sizes, inferring the impact dependent on the type and concentration of dye solutions on the physiological growth and development processes. Dunn post hoc tests showed significant differences, especially in the case of comparisons between treated and control batches, but also between the two concentrations of the same dye, especially with regard to green and turquoise dyes. In the case of biomass growth and accumulation parameters, a relationship dependent on the type and concentration of the dye solution was observed, with the red and turquoise dye solutions generating more pronounced effects on biomass dynamics and mineral matter accumulation, while the green dye produced statistically significant changes even at lower concentrations, indicating an early influence on physiological processes. Spearman correlations demonstrated strong intra-lot associations between gravimetric parameters, suggesting their interdependence, as well as the presence of specific disturbances on the water, organic and mineral balance induced by the applied treatments, compared to the results of the control lot that denoted physiological stability in the absence of chemical stress, through the reduced number of statistically significant correlations. At the physiological level, growth parameters (RGR) indicated an effect dependent on the type of dye solution, but independent of concentration, especially for the red and turquoise dyes. Mineral deposition and tissue composition (TDM, MC, OC, OC/MC) implied metabolic imbalances, suggesting disturbances in ionic homeostasis and biosynthetic processes. Tissue density (TD) showed a systematic decrease, correlated with water accumulation, while water parameters (WC, S, WC/DW) denoted the existence of compensatory mechanisms associated with osmotic stress, especially in the case of the green dye solutions. Stress indicators (GI, STI, TOI) and biomass accumulation efficiency factor (E) underlined the adaptive nature of the biological response, especially in the case of the red dye solutions.

The results obtained in the experiment are in accordance with the trends reported in the specialized literature on the phytotoxic and cytotoxic effects of food dyes, highlighted by the *Allium cepa* test. Thus, the significant reduction in the mitotic index described by Leme and Marin-Morales [10] and Adesuyi et al. [70] in *Allium cepa* root assay, and Kalsoom et al. [71] on *Allium sativum*, correlated with the increase in the frequency of chromosomal aberrations, could be indirectly associated with the gravimetric and physiological changes observed in the present study. In particular, the decrease in biomass accumulation efficiency, the increase in the growth inhibition index and the differentiated values of the tolerance indices reflect a decrease in the processes of cell division and expansion, a plausible consequence of the mitodepressive effect described for dyes such as Sunset Yellow (E110), Carmoisine (E122) and Brilliant Blue (E133) [19,33]. Similar studies conducted on *Allium cepa* have revealed a dose-dependent inhibitory effect on the physiological parameters of cell proliferation, exerted by Brilliant Blue [72,73]. Mutagenicity and inhibition of cell proliferation parameters were also observed in studies conducted on *Trachyspermum ammi* and *Vicia faba*, in which Tartrazine (E102), Sunset Yellow and Quinoline Yellow (E104) demonstrated statistically significant concentration-dependent effects [74,75].

Moreover, the significant imbalances observed in mineral content, OC/MC ratio and tissue mineral deposition could be correlated with the disturbances in ionic homeostasis and cellular metabolism mentioned by Khan et al. [76], suggesting that the mechanisms of toxicity involve not only the inhibition of mitosis, but also the alteration of ion transport and accumulation, similar results being observed in studies carried out on aquatic organisms such as *Lemna minor* [77]. In the same sense, changes in water parameters and decreased tissue density indicate an adaptive response to stress, comparable to the physiological imbalances reported in studies on *Brassica campestris*, *Vicia faba*, *Cucumis sativus* and *Zea mays*, where exposure to dyes, such as Tartrazine, Quinolein Yellow and Sunset Yellow, caused disturbances in water balance and oxidative metabolism [19,33,42,45,76,78,79,80].

In addition, the growth inhibition trend and the modification of development rates observed experimentally are consistent with the results obtained on *Triticum aestivum* and *Lemna minor*, where Tartrazine and Brilliant Blue respectively produced concentration-dependent effects on root elongation [81] and significant relative growth rate inhibition (up to 33%) dependent on the parameter used to evaluate the physiological response [82]. Thus, the comparison of the present and available data suggests that the effects observed at the biomass and physiological parameters level plausibly describe the integrated expression of cytotoxic and genotoxic processes manifested by the inhibition of cell division, disruption of the mitotic spindle and induction of metabolic stress.

## 4. Materials and Methods

### 4.1. Determination of the Applied Treatments

Synthetic food dyes, especially azo dyes, are often found in home cooking or artisanal confectioneries, used for the same purposes as in industrial processes, where they appear in the form of gels, water-soluble and fat-soluble powders, markers or sprays, intended for decorating cakes, icings, creams, candies, jellies, ice cream and drinks [42,83].

These concentrated forms are favored because they allow for the achievement of intense shades using a small amount of product, without diluting the compositions in which they are added, dispersing well both in aqueous matrices (icings, syrups, gelatins) and in fat-rich masses (doughs, butter-based creams, chocolate), offering good stability to light, pH and heat treatments, so that the product can maintain its aesthetic appearance for a long time [59,83]. The high coloring power, the very low cost and the absence of any additional taste or odor make these colorant sets easy to use in an empirical manner, without taking into account the recommended administration doses [41,45,84].

In order to assess the potential physiological effects associated with exposure to commercial food dye formulations used at their recommended application levels, three commercially available gel-based food dyes were selected. These products, which can be purchased and used without restrictions, contained variable proportions of Tartrazine (E102), Quinoline Yellow (E104), Sunset Yellow (E110), Carmoisine (E122), and Brilliant Blue (E133). The complete composition of the tested products is presented in Table 4.

### 4.2. Experimental Design

Considering a standard for rapid assessment of toxicity and environmental pollution levels, *Allium cepa* was chosen as a model organism in the present study, being one of the most widely used biological indicators for assessing the cytogenotoxicity of chemical compounds, including food dyes [10,70]. In this case, the *Allium* test was used to determine the phytotoxicity of synthetic dyes by monitoring bulb growth parameters.

A total of 105 *Allium cepa* var. *cepa*, cultivar Sturon (yellow onion) bulbs of visually similar size were used in the experiment, previously selected to avoid possible signs of disease or growth defects. The dried cataphyllum and possible traces of inorganic matter were removed from the surface of the bulbs, which were subsequently divided into 2 groups of 45 bulbs and one of 15 bulbs, corresponding to the two different concentrations of the tested dyes—each group being then distributed into three batches of 15 bulbs, one for each dye—and, respectively, the control batch. The bulbs were placed in approximately 2 mL Eppendorf tubes filled with tap water, with only the basal root disk immersed in the aqueous medium. The tubes were mounted on a perforated polystyrene board and secured at the cap level with wooden picks, ensuring their stable vertical orientation during the entire experiment. They were left in water for 4 days to germinate and form a new root network. The water volume was supplemented and refreshed periodically to ensure permanent contact and to prevent possible microbial contamination.

Based on each chosen dye, two solutions with predetermined concentrations were prepared, representing the maximum recommended dose/kg (concentration 1) for each gel in turn and, respectively, half of this dose or half dilution of the initial solution (concentration 2). For each gel, the maximum amount recommended by the manufacturer was weighed (6.8 g for the red dye gel, 2.4 g for the turquoise dye gel, and 0.99 g for the green dye gel), then dissolved in one liter of water from the public distribution network, thus obtaining the solution of concentration 1 (with a value of 6.8% for the red dye, 2.4% for the turquoise dye, and 0.99% for the green dye). Half of its volume was transferred to another container, to which 500 mL of tap water was added, thus obtaining the solution of concentration 2 (with a value of 3.4% for the red dye, 1.2% for the turquoise dye, and 0.495% for the green dye). The exact concentration values of the 6 tested solutions can also be found in Table 4.

The treatments were applied for 48 h by replacing the water with the 2 solutions of different concentrations of each dye, during which the roots of the bulbs were in permanent and direct contact with the assigned solution.

### 4.3. Gravimetric Measurements

The experiment was carried out over a period of 6 days, at the end of which the initial wet biomass, pre-treatment wet biomass, post-treatment wet biomass, dry biomass and ash of the bulbs grown under hydroponic conditions were determined, all measurements being made using a high-precision analytical balance (Kern & Sohn GmbH, model ABT 220-5DNM, Germany). At the same time, the root length, the growth and development of the plant mass and any visible morphological changes that occurred along the way were observed.

The initial fresh biomass was obtained on the first day of the experiment, at the time of bulb selection. The pre-treatment wet biomass was determined on the 4th day of the experiment, following the appearance of adventitious roots, the bulbs being removed from the installation and blotted with a paper towel, so as to remove as much water as possible from the root surface. Post-treatment wet biomass was obtained 48 h after the treatments were applied, in a similar manner to the previous measurement. Subsequently, the bulbs were oven-dried (Memmert GmbH, model UF55, Germany) at 90 °C for 14 h and weighed after cooling to determine the dry biomass. The plant material was burned in the calcination furnace (Nabertherm GmbH, model L3/11, Germany) at 500 °C for 2 h, and the ash obtained was then weighed.

In order to evaluate the physiological response of plant organisms to the applied treatments, 13 parameters derived from the 5 gravimetric measurements were determined, in order to characterize the changes in tissue composition, water status, growth and stress tolerance:

- *The organic content* relative to the wet biomass after treatment (OC = (DW − AC)/FW × 100, %), representing the biologically active fraction of the biomass [85];
- *The mineral content* relative to the wet biomass after treatment (MC = AC/FW × 100, %), indicating the degree of inorganic loading of the tissues [85];
- *The organic/mineral ratio* (OC/MC) to highlight the balance between organic and inorganic components;
- *Tissue dry mineral content* (TDM = AC/DW × 1000, g/kg), illustrates the amount of inorganic compounds accumulated in the dry mass [85];
- *Tissue density* (TD = DW/FW × 1000, g/kg) reflects the proportion of dry matter in the fresh biomass [86].

The water parameters included were:

- *Succulence* (S = (FW − DW)/(DW − AC)), which expresses the amount of water reported to the organic mass [85];
- *Water content* (WC = (FW − DW)/FW × 100, %), relative to the post-treatment wet biomass, representing the proportion of water in the tissues [87];
- *The water/dry matter ratio* (WC/DW), which indicates the level of hydration relative to the solid fraction.

To evaluate the effects on growth and tolerance, the following parameters were calculated:

- *The growth inhibition index* (GI = (mWc − Wt)/mWc × 100, %), which expresses the reduction in growth compared to the control [88];
- *The tolerance index* (TOI = DWt/DWc × 100, %), which reflects the ability of plants to maintain growth under stress conditions [89];
- *The stress tolerance factor* (STI = (Wt × Wc)/mWc), used to compare the relative performance of the treated lots [90].

In addition, *the biomass accumulation efficiency* (E = (Wpost − Wpre)/Wpre × 100, %) [82] was used to describe the growth capacity in the experimental interval, and *the relative growth rate* (RGR = (lnW_2_ − lnW_1_)/(t_2_ − t_1_)) [91] was used to evaluate the dynamics of biomass accumulation over time.

In these relationships, FW represents the wet biomass, DW the dry biomass, AC the ash content, Wc the biomass of the control batch, Wt the biomass of the treated batch, mWc the average biomass of the control batch, and Wpre and Wpost the biomasses determined before and after the application of the treatments. Regarding the relative growth rate, for RGR, pre-W1 was considered the initial biomass, and W_2_ the pre-treatment fresh biomass, t_1_ being day 0, and t_2_ day 4 (the difference obtained being 4). Similarly, for RGRpost, W_1_ was considered the pre-treatment fresh biomass, and W_2_ the post-treatment fresh biomass, t_1_ being day 4, and t_2_ day 6 (the difference obtained being 2).

### 4.4. Statistical Analysis

At the end of the experimental period, the raw data were initially recorded and processed using Microsoft Office Excel (v2018), and then statistically analyzed using PAST software (v4.03) [92].

The distribution of the data was analyzed using the Shapiro–Wilk normality test, and the analysis of variance of the gravimetric data and those obtained from the calculation of physiological parameters was performed using the Kruskal-Wallis test. Values of *p* < 0.05 were considered statistically significant. The Dunn test was used post hoc, in order to identify the differences between specific pairs of groups, following a significant result of the Kruskal–Wallis test. The intra-lot relationship of the gravimetric parameters was analyzed by determining Spearman’s rank correlation coefficient.

## 5. Conclusions

The *Allium cepa* test represents a standard in genotoxicity and mutagenicity evaluations due to its simplicity and short application time, good correlation with other test systems and high efficiency, being one of the most reliable biomonitoring tests for various xenobiotics, including food dyes. In the case of the present study, the use of gravimetric and physiological parameters was pursued, to the detriment of the commonly used genetic ones, in order to monitor the phytotoxic effects induced by mixtures of synthetic food dyes.

The experimental data obtained in the present study indicate that the exposure of plant organisms grown under hydroponic conditions to solutions containing different concentrations of food dyes could significantly influence the growth parameters and gravimetric characteristics analyzed. Statistical analyses revealed the existence of significant differences between the experimental and control groups for several of the evaluated parameters, suggesting that these substances can potentially exert detectable phytotoxic effects on plant biological systems. The variability of the response observed between the different types of dye solutions and between the concentrations tested could also indicate that the biological effects of these compounds depend on the chemical nature of the dye, but not on the level of exposure in the vast majority of the analyzed cases.

Nevertheless, given the complex composition of the commercial food dye gels used in the present study, which included not only colorants but also thickening agents, preservatives, pH regulators, and solvents, the observed effects cannot be unequivocally ascribed to the food dyes alone, as the influence of the accompanying additives remains unknown. In addition, the study focused exclusively on gravimetric and physiological endpoints. Future investigations incorporating biochemical and cytogenetic or molecular analyses would contribute to a more comprehensive characterization of the mechanisms involved and to a more robust evaluation of the phytotoxic potential of these formulations. Furthermore, considering that synthetic food dyes are expected to reach groundwater systems in highly diluted concentrations following environmental release and dispersion, future studies should also evaluate substantially lower concentrations at longer-term exposures than those employed in the present experiment. Such an approach would provide a more realistic assessment of environmentally relevant exposure scenarios and improve the ecological relevance of the findings.

The results obtained reiterate the usefulness of the *Allium cepa* test as a sensitive, rapid and relevant method for the evaluation of the phytotoxic effects of synthetic food dyes. At the same time, it is emphasized that, although considered safe under the regulated conditions of use in the food industry, synthetic food dyes can exhibit relevant biological effects when they reach the natural environment in higher concentrations or in complex combinations. However, it is still necessary to evaluate the cumulative effects and exposure to complex mixtures of chemical additives on the environment and living organisms, in order to develop more effective strategies for contamination management and reducing anthropogenic pressure on natural ecosystems.

## Figures and Tables

**Table 1:** Descriptive statistics for gravimetric parameters.

		Min.	Max.	Mean	Median	Q1	Q3	IQR	Str. Dev.	Coef. Var.	Skewness	Kurtosis
**FWi**	**C1R**	3.41929	5.64742	4.28555	4.19479	3.69185	4.80973	1.11788	0.69928	16.31708	0.40159	−0.98614
	**C1T**	1.08251	2.07512	1.43005	1.28406	1.16880	1.68484	0.51604	0.32985	23.06551	0.67229	−0.92165
	**C1G**	1.41636	3.10694	2.22582	2.20692	1.82068	2.62838	0.80770	0.52990	23.80703	0.10296	−1.23091
	**C2R**	3.41794	6.28681	4.62842	4.15108	3.97995	5.30252	1.32258	0.88643	19.15185	0.40338	−1.06913
	**C2T**	0.73027	1.50821	1.12556	1.17588	0.95072	1.24219	0.29147	0.22792	20.24936	−0.32587	−0.64423
	**C2G**	1.50958	3.33343	2.24405	2.07542	1.77238	2.64838	0.87600	0.59243	26.39988	0.76183	−0.61047
	**Control**	2.18437	3.18581	2.53709	2.52124	2.40716	2.59049	0.18333	0.26179	10.31847	0.95514	1.60301
**FWpre**	**C1R**	3.70769	6.60465	5.12250	5.07377	4.59012	5.67677	1.08665	0.78081	15.24269	0.21548	−0.37593
	**C1T**	1.34741	2.43587	1.81266	1.58743	1.51519	2.10081	0.58562	0.38984	21.50663	0.30408	−1.56476
	**C1G**	1.98766	4.07319	3.03611	2.92900	2.58773	3.54936	0.96164	0.65905	21.70698	0.02071	−1.06076
	**C2R**	4.09581	7.05098	5.56412	5.47993	4.79184	6.48671	1.69487	0.95932	17.24112	0.25746	−1.35566
	**C2T**	0.84175	1.84712	1.39079	1.44833	1.24736	1.57135	0.32400	0.30911	22.22568	−0.49440	−0.39920
	**C2G**	1.72302	4.22925	2.80772	2.58829	2.39775	3.27804	0.88029	0.65721	23.40712	0.58608	0.21103
	**Control**	2.61292	3.91353	3.08635	3.04537	2.85637	3.21249	0.35613	0.35083	11.36725	1.07753	1.10567
**FWpost**	**C1R**	3.85342	7.40690	5.47064	5.22462	4.95862	5.74289	0.78427	0.91119	16.65603	0.63693	0.48022
	**C1T**	1.35811	2.75499	2.00690	1.86283	1.66291	2.33286	0.66996	0.43738	21.79402	0.23336	−1.29684
	**C1G**	2.12004	4.30800	3.39383	3.51225	2.84078	3.98602	1.14524	0.70912	20.89429	−0.27576	−1.13189
	**C2R**	4.09827	7.71566	6.09268	6.00405	5.43548	7.19593	1.76045	1.10219	18.09034	−0.00121	−0.91167
	**C2T**	0.85952	2.12561	1.55497	1.55134	1.33871	1.81746	0.47875	0.37823	24.32400	−0.30692	−0.44760
	**C2G**	1.92608	4.70666	3.15863	2.93590	2.77856	3.67001	0.89146	0.70491	22.31704	0.61614	0.65195
	**Control**	2.81261	4.46045	3.40734	3.25777	3.08627	3.61607	0.52980	0.46227	13.56689	0.99680	0.57342
**DW**	**C1R**	0.48122	1.07333	0.66310	0.63091	0.54773	0.71805	0.17032	0.15563	23.47056	1.39425	2.21013
	**C1T**	0.13544	0.37480	0.23137	0.23012	0.18815	0.27159	0.08344	0.06143	26.54879	0.65391	0.68899
	**C1G**	0.20763	0.52558	0.32858	0.31547	0.25131	0.36712	0.11581	0.09727	29.60148	0.62775	−0.41272
	**C2R**	0.47836	1.07246	0.78227	0.82318	0.63571	0.93128	0.29557	0.18491	23.63710	−0.23744	−1.11136
	**C2T**	0.13015	0.27641	0.18734	0.18352	0.16114	0.20897	0.04784	0.03834	20.46475	0.84509	0.55924
	**C2G**	0.20667	0.51687	0.33125	0.27899	0.25380	0.41744	0.16364	0.10365	31.29020	0.66862	−0.98303
	**Control**	0.32287	0.54673	0.39556	0.37987	0.34698	0.42547	0.07849	0.06165	15.58515	1.04546	1.08354
**AC**	**C1R**	0.03421	0.06068	0.04661	0.04543	0.03974	0.05402	0.01428	0.00882	18.92699	0.32539	−1.15662
	**C1T**	0.01217	0.03267	0.02216	0.02172	0.01888	0.02525	0.00637	0.00537	24.22310	0.28908	−0.00567
	**C1G**	0.01685	0.03661	0.02578	0.02626	0.02140	0.02983	0.00843	0.00602	23.35101	0.28222	−0.77918
	**C2R**	0.03660	0.07012	0.05084	0.05127	0.04273	0.05843	0.01571	0.01028	20.21062	0.19940	−1.00409
	**C2T**	0.01285	0.05134	0.03074	0.02830	0.01989	0.04495	0.02506	0.01392	45.27524	0.32960	−1.38821
	**C2G**	0.01595	0.04465	0.02571	0.02424	0.02078	0.02893	0.00815	0.00766	29.80188	1.30305	1.71414
	**Control**	0.02565	0.03929	0.03280	0.03161	0.02988	0.03560	0.00572	0.00397	12.09499	0.06310	−0.73438

Note: the parameters discussed were abbreviated as initial fresh biomass (FWi), pre-treatment fresh biomass (FWpre), post-treatment fresh biomass (FWpost), dry biomass (DW) and ash content (AC). The analyzed batches were abbreviated according to the concentration and type of dye applied, as follows: C1R represents the lot treated with concentration 1 of the red dye solution, C2R represents the lot treated with concentration 2 of the red dye solution, C1T represents the lot treated with concentration 1 of the turquise dye solution, C2T represents the lot treated with concentration 2 of the turquise dye solution, C1G represents the lot treated with concentration 1 of the green dye solution, C2G represents the lot treated with concentration 2 of the green dye solution, and the control represents the batch treated with tap water used as a negative control group.

**Table 2:** Kruskal–Wallis test results for gravimetric parameters.

	H	*p*	ε^2^
**FWi**	88.69	5.669 × 10^−17^	0.83186
**FWpre**	88.84	5.285 × 10^−17^	0.83340
**FWpost**	87.42	1.041 × 10^−16^	0.82729
**DW**	82.79	9.489 × 10^−16^	0.77906
**AC**	60.35	3.824 × 10^−11^	0.54531

Note: H represents the test statistic, *p* represents the probability value and epsilon squared (ε^2^) represents the effect size. For *p*-values < 0.05, data are considered statistically significant. The significance levels considered for the effect size are small effect (0.01–0.06), moderate effect (0.06–0.14), and large effect (>0.14).

**Table 3:** Results of the Kruskal–Wallis test on physiological parameters.

	H	*p*	ε^2^
**RGRpre**	19.71	0.003118	0.120721649
**RGRpost**	86.45	1.654 × 10^−16^	0.8171875
**TDM**	47.76	1.317 × 10^−8^	0.414166667
**TDi**	21.08	0.001774	0.13625
**TDpre**	23.1	0.0007634	0.157291667
**TDpost**	21.68	0.001381	0.1425
**MC**	46.09	2.843 × 10^−8^	0.396770833
**OC**	20.22	0.002531	0.127291667
**OC/MC**	47.76	1.317 × 10^−8^	0.414166667
**WC**	21.68	0.001381	0.141030928
**S**	20.17	0.002584	0.125463918
**WC/DW**	80.92	2.311 × 10^−15^	0.751752577
**E**	14.57	0.0239	0.0684375
**GI**	69.81	1.121 × 10^−13^	0.76597561
**STI**	63.33	2.49 × 10^−12^	0.68695122
**TOI**	67.63	3.19 × 10^−13^	0.739390244

Note: For *p*-values < 0.05, data are considered statistically significant. The significance levels considered for the effect size are small effect (0.01–0.06), moderate effect (0.06–0.14), and large effect (>0.14).

**Table 4:** List of constituent ingredients of the 3 food dyes tested.

Selected Synthetic Food Dye	Ingredients	Maximum Recommended Dose	Tested Concentration Values	
			Concentration 1	Concentration 2
Dye 1 “Christmas Red”	glycerol (E422), glucose syrup, water, carmoisine (E122), sunset yellow FCF (110), tartrazine (E102), modified starch (E1422), xanthan gum (E415), potassium sorbate (E202), citric acid (E330)	6.8 g/kg	6.8%	3.4%
Dye 2 “Turquoise”	glycerol (E422), glucose syrup, water, brilliant blue FCF (E133), quinoline yellow (E104), modified starch (E1422), xanthan gum (E415), potassium sorbate (E202), citric acid (E330)	2.4 g/kg	2.4%	1.2%
Dye 3 “Grass Green”	glycerol (E422), glucose syrup, water, tatrazine (E102), brilliant blue FCF (E133), sunset yellow FCF (E110), modified starch (E1422), xanthan gum (E415), potassium sorbate (E202), citric acid (E330)	0.99 g/kg	0.99%	0.495%

## Data Availability

The original contributions presented in this study are included in the article. Further inquiries can be directed to the corresponding author.

## Abbreviations

The following abbreviations are used in this manuscript:

AC	Ash content
C1	Concentration 1 of the dye solutions
C2	Concentration 2 of the dye solutions
C1G	The lot treated with concentration 1 of the green dye solution
C2G	The lot treated with concentration 2 of the green dye solution
C1R	The lot treated with concentration 1 of the red dye solution
C2R	The lot treated with concentration 2 of the red dye solution
C1T	The lot treated with concentration 1 of the turquoise dye solution
C2T	The lot treated with concentration 2 of the turquoise dye solution
Control	The lot treated with tap water and used as a negative control group
DW	Dry biomass
E	Biomass accumulation efficiency
FWi	Initial fresh biomass
FWpost	Post-treatment biomass
FWpre	Pre-treatment biomass
GI	Growth inhibition
MC	Mineral content
OC	Organic content
RGR	Relative growth rate
S	Succulence
STI	Stress tolerance index
TD	Tissue density
TDM	Tissue dry mineral content
TOI	Tolerance index
WC	Water content
